# Refinement and Rescoring of Virtual Screening Results

**DOI:** 10.3389/fchem.2019.00498

**Published:** 2019-07-11

**Authors:** Giulio Rastelli, Luca Pinzi

**Affiliations:** Department of Life Sciences, University of Modena and Reggio Emilia, Modena, Italy

**Keywords:** docking, post-docking, virtual screening, molecular dynamics, BEAR, binding free energy

## Abstract

High-throughput docking is an established computational screening approach in drug design. This methodology enables a rapid identification of biologically active hit compounds, providing an efficient and cost-effective complement or alternative to experimental high-throughput screenings. However, limitations inherent to the methodology make docking results inevitably approximate. Two major Achille's heels include the use of approximated scoring functions and the limited sampling of the ligand-target complexes. Therefore, docking results require careful evaluation and further post-docking analyses. In this article, we will overview our approach to post-docking analysis in virtual screenings. BEAR (Binding Estimation After Refinement) was developed as a post-docking processing tool that refines docking poses by means of molecular dynamics (MD) and then rescores the ligands based on more accurate scoring functions (MM-PB(GB)SA). The tool has been validated and used prospectively in drug discovery applications. Future directions regarding refinement and rescoring in virtual screening are discussed.

## Introduction

High-throughput screening (HTS) is a widely used method for the discovery of biologically active hits. However, the high costs and the low hit rates characterizing such experiments often make HTS not affordable for academic labs or small companies (Sliwoski et al., 2014). As a consequence, high-throughput docking screenings represent an attractive alternative (Irwin and Shoichet, 2016). Structure-based virtual screenings (SBVSs) require the knowledge of the three-dimensional structure of the target of interest, as well as the access to large libraries of small molecules available in public databases (Kar and Roy, 2013; Rastelli, 2013). Docking programs generate binding poses of compounds in the active site of a target and evaluate the ligand binding strength by means of scoring functions (Lengauer and Rarey, 1996; Kitchen et al., 2004). Several docking software relying on different algorithms have been developed for virtual screening so far (Rarey et al., 1996; Morris et al., 1998; Friesner et al., 2004; Sánchez-Linares et al., 2012). However, although remarkable improvements have been obtained along the years, several drawbacks and limitations still exist (Huang and Zou, 2010; Rastelli, 2013). First of all, sampling the conformational space accessible to ligand-target complexes in an induced-fit context is a difficult and target-dependent task. To help overcoming such limitations, several *in silico* strategies including molecular dynamics or induced fit strategies have been introduced (Sherman et al., 2006; Nabuurs et al., 2007; Caporuscio and Rastelli, 2016). Secondly, docking scores and experimental binding affinities usually do not correlate, because screening large numbers of compounds in a reasonable time requires the use of approximate scoring functions. Together, the two effects imply that a variable number of false-positive and false-negative hits populate the ranked lists, which then require careful evaluation and further post-docking analyses. Hence, it has become general opinion that docking results should be improved by means of more rigorous post-docking processing strategies. Several post-processing strategies have been developed to overcome docking limitations over the past decades. In particular, methods based on binding free energy estimations have demonstrated to provide higher hit rates and to be more suitable for ranking cognate ligands in virtual screening (Hou et al., 2011; Genheden and Ryde, 2015; Pu et al., 2017), the predicted binding free energy usually correlating better with experimental data (Brandsdal et al., 2003). One of the first reported energy-based methods is MM-PB(GB)SA, which was developed to more accurately assess the relative free energy of binding for a given macromolecular system from molecular dynamics simulations (Kollman et al., 2000). This method represented a remarkable step forward to the obtainment of *in silico* predicted binding affinities that are in good agreement with experiments. In fact, it was extensively used to evaluate the free energy of binding for a number of complexes in the last years (Gohlke et al., 2003; Hou and Yu, 2007; Ferri et al., 2009; Yang et al., 2012). For example, it was successfully used for identifying residue *hot-spots* outside the binding interface of the Ras–Raf and Ras–RalGDS protein-protein complexes, discussing also their implications for an allosteric activation of the proteins (Gohlke et al., 2003). More recently, this method was also employed for predicting binding affinities of few inhibitors of HIV-1 protease and to help rationalize drug resistance caused by the mutations on the enzyme binding site (Hou and Yu, 2007). However, it should also be noted that MM-PB(GB)SA results are dependent on the employed parameters and receptor structures used in the calculations (Xu et al., 2013; Sun et al., 2014, 2018; Genheden and Ryde, 2015). More accurate free energy-based methods have also been reported (Brandsdal et al., 2003; Jorgensen and Thomas, 2008; Parenti and Rastelli, 2012; Limongelli et al., 2013; De Vivo et al., 2016). Among them, it is worth mentioning the Free Energy Perturbation (FEP) method, which allows to estimate the free energy of binding of a ligand to a protein by decomposing the system through a series of “alchemical transformations” (Jorgensen and Thomas, 2008; De Vivo et al., 2016). More recently, funnel-metadynamics (FM) methods that use a funnel-shaped potential limiting the sampling space available for a ligand to bind/unbind to a protein have been proposed (Limongelli et al., 2013; De Vivo et al., 2016). However, although these methods demonstrated to accurately estimate ligand binding, they are time-consuming and therefore not suitable for virtual screening rescoring of large databases. With the aim of improving ligand-binding estimations of docking complexes at reasonable computational costs, we developed Binding Estimation After Refinement (BEAR) (Rastelli et al., 2009). BEAR is an automated post-docking tool based on conformational refinement of docking poses with molecular dynamics followed by a more accurate prediction of binding free energies performed with MM-PBSA and MM-GBSA, which take into account desolvation energies (Kuhn et al., 2005; Lyne et al., 2006; Rastelli et al., 2010; Genheden and Ryde, 2015). As it allows accurately rescoring docking poses in reasonable times, BEAR can be considered an efficient tool that could be routinely used for virtual screening. In this article, we will briefly describe the BEAR tool, providing an overview of the validation studies performed so far. Finally, we will describe its prospective applications in drug discovery campaigns and comment on future directions of refinement and rescoring methods.

## The BEAR Tool

**Figure 1:**
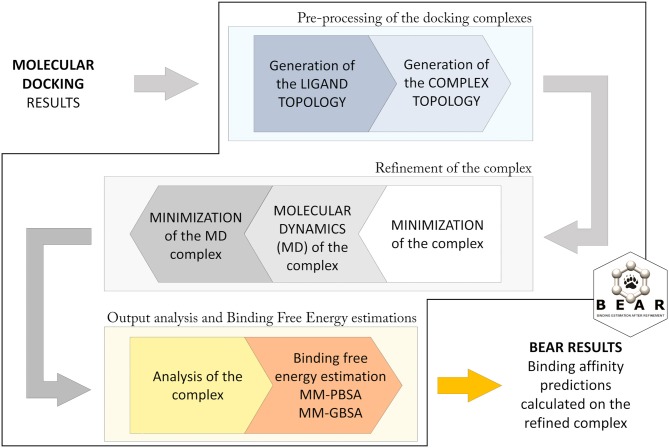
Computational workflow applied in BEAR.

The BEAR workflow (Figure 1; Rastelli et al., 2009) consists of an initial pre-processing step in which hydrogen atoms are added to the protein, atomic charges (AM1-BCC) are calculated for the docked molecules, and missing force-field parameters are assigned. Then, topologies for the ligand, the protein, and the ligand-protein complex are built. In particular, ligand atom types are assigned according to the Generalized Amber Force Field (GAFF) (Wang et al., 2004), while, the atom types and charges of amino acids are assigned according to the Amber ff03 force field (Duan et al., 2003). The following iterative three steps procedure is based on molecular mechanics (MM) and molecular dynamics (MD) cycles. In particular, an initial MM energy minimization of the whole protein–ligand complex is performed, followed by a short MD simulation where the ligand is allowed to move, and a final re-minimization of the entire complex. All the minimization tasks are performed through 2000 steps without restraints, and with a distance-dependent dielectric constant ε = 4r and a cutoff of 12 Å. The MD simulation is performed at 300 K for 100 ps, with the SHAKE parameter turned on and a time-step of 2.0 fs. This protocol allows evaluating the reliability of the predicted docking complex and to establish potential additional ligand-protein interactions resulting from the structural refinement of the complex, thus obtaining more accurate binding energy predictions. After refinement of the complex, the free energy of binding of the ligand is calculated with the MM-PBSA and MM-GBSA methods. These operations are implemented with the use of AMBER modules (Case et al., 2018). Further details about the BEAR tool are described in Rastelli et al. (2009).

## Benchmarking Studies

The post-docking tool BEAR has been extensively validated in various test cases. First of all, the MM/MD protocol described above was investigated on a series of aldose reductase inhibitors with notable chemical diversity. Remarkably, the calculated free energies of binding after refinement of ligand-protein complexes resulted to be highly correlated with experimental affinities. This study demonstrated that different classes of aldose reductase inhibitors could be accurately rescored with our procedure (Ferrari et al., 2007). Extensive validations were also made on *Plasmodium falciparium* dihydrofolate reductase (PfDHFR). These simulations aimed at evaluating the performance of BEAR in virtual screening settings of different size and complexity. Firstly, BEAR performed well in discriminating 14 known inhibitors of PfDHFR from the 1,720 compounds included in the National Cancer Institute diversity database (Rastelli et al., 2009). The achieved performances were clearly superior to those of AutoDock (Morris et al., 1998), demonstrating that rescoring of the predicted docking poses with BEAR heavily improved SBVS results. In a second experiment, enrichment factors (EFs) obtained with BEAR were evaluated by seeding 201 known inhibitors with 7,150 decoys as contained in the DHFR data set of the Directory of Useful Decoys database (Mysinger et al., 2012). Moreover, the same set of ligands was also seeded into the 1.5 million compounds belonging to the lead-like subset of the ZINC database (Irwin et al., 2012), this latter benchmark reflecting a typical virtual screening setting. In both cases, BEAR refinement and rescoring yielded significantly higher EFs compared to docking (Degliesposti et al., 2011). This was also an opportunity for fine-tuning the BEAR parameters, and thus achieving good performances at reasonable computational costs.

The BEAR performance was also assessed on biological targets characterized by flexible binding sites and/or containing water molecules in the binding pocket. Such targets are particularly challenging for SBVS (Elokely and Doerksen, 2013). In fact, certain ligand chemotypes could fit with favorable scores into certain protein conformations but not in others, thus hampering their identification in a virtual screening. To evaluate whether docking into multiple protein conformations (ensemble docking) instead of using a single representative structure would improve BEAR predictions for “difficult” targets (Sgobba et al., 2012), we investigated targets of different families (adenosine deaminase, factor Xa, estrogen receptor, thymidine kinase, aldose reductase, and enoyl ACP reductase). Interestingly, a comparative analysis of the EFs obtained for different proteins and multiple protein conformations revealed that the application of BEAR was able in several cases to yield higher EFs compared to docking. However, in challenging targets such as adenosine deaminase and enoyl ACP reductase, all scoring functions failed in yielding high EFs. This effect was attributed to difficulties in predicting correct ligand binding modes in these two targets. In particular, when the docked pose was completely wrong, for example head-to-tail with respect to the correct binding mode, the MM/MD refinement stage was not enough to turn the binding mode into the correct one. Therefore, the advantage of using MM-PBSA and MM-GBSA in prioritizing active compounds is dependent on the obtainment of correct binding modes, which makes the refinement and rescoring procedures intimately connected.

More recently, BEAR was also applied to screen ligands of G-protein coupled receptors (GPCRs) with known crystal structure, namely β_2_-adrenergic (β_2_), adenosine A_2A_ (A_2A_), dopamine D_3_ (D_3_), and histamine H_1_ (H_1_) receptors (Anighoro and Rastelli, 2013). Results were analyzed in terms of the ability to recognize known antagonists from decoys, as well as to predict correct binding modes. In all cases except for A_2A_, significant or dramatic improvements of EFs were obtained after the application of BEAR. A_2A_ was challenging because antagonists participated to an extended water-mediated hydrogen bond network. Interestingly, explicit consideration of a suitable number of these structural waters significantly improved the predictions. This finding is in line with the fact that MM-PB(GB)SA calculations do not explicitly consider water molecules mediating ligand-protein interactions, and binding mode predictions heavily depend on the presence of bridging water molecules participating to hydrogen bond networks. For all GPCRs, a more accurate account of desolvation effects, such as the one performed by MM-PBSA, is important to accurately predict the affinity of the protonated biogenic amines. We also found that five known H_1_ and D_3_ receptor antagonists were top-scored and ranked well in each of the two target screenings, prospecting for the first time the utility of post-docking tools in multi-target drug design (Anighoro et al., 2014). Indeed, as the free energy of binding calculated by BEAR allows to more accurately predicting the affinity of ligands for their target(s) with a reasonable computer time investment, we envision that *in silico* strategies embedding this tool can be useful to allow the identification of ligands with the desired multi-target profiles.

## Prospective Validations

The BEAR workflow has also been implemented in the computing GRID infrastructure EGEE, as part of the WISDOM (Wide *in silico* DOcking on Malaria) initiative against malaria (Kasam et al., 2009). Then, it was deployed to perform virtual screenings against antimalarial drug targets. One massive data challenge was performed on Plasmepsin II, an aspartic protease involved in the metabolism of *P. falciparum* (Degliesposti et al., 2009). In this work, BEAR was used to refine and rescore the 5,000 top-scoring compounds docked with FlexX (Rarey et al., 1996). Then, the final step of candidates' selection was performed on the top 200 compounds resulting from both MM-PBSA and MM-GBSA ranked lists. Interestingly, an analysis of the BEAR ranked lists, together with an inspection of the protein-ligand complexes and a similarity-based clustering of the ligands allowed selecting 30 compounds belonging to 5 different chemotypes as potential Plasmepsin II inhibitors. Remarkably, 26 of them were active, resulting in an impressive hit rate of 87%, and some of the compounds displayed nanomolar inhibitory activity.

More recently, BEAR was successfully applied in a virtual screening campaign that allowed the identification of the *first-in-class* allosteric inhibitors of CDK2 (Rastelli et al., 2014). In this work, around 600.000 commercially available compounds were screened against a crystal structure of CDK2 with an open type III allosteric pocket, by using AutoDock for docking and BEAR for post-docking analyses. The adopted virtual screening protocol led to the identification of 7 allosteric ligands of CDK2, providing a hit rate of 20%. Interestingly, the most potent compound was able to selectively inhibit CDK2-mediated Retinoblastoma phosphorylation, confirming that its mechanism of action is fully compatible with a selective inhibition of CDK2 phosphorylation in cells. Moreover, some of these ligands inhibited the proliferation of MDA-MB231 and ZR-75-1 breast cancer cells with IC_50_ values in the low micromolar range (Rastelli et al., 2014).

## Final Remarks

Although many progresses have been made in molecular docking, limitations deriving from the use of rigid protein conformations and of approximate scoring functions often impair virtual screening results. Therefore, docking results require careful evaluation and further post-docking analyses. BEAR is a post-processing tool that performs binding free energy estimations after MM and MD refinement of docking complexes. Our previous studies demonstrated that BEAR performed well in a number of benchmarking investigations, as well as in discovering biologically active hits in different prospective virtual screening campaigns. Moreover, as it not computationally demanding as other free energy-based methods, it constitutes a reasonable compromise to obtain accurate rescoring of ligands at reasonable computational costs. One might argue that the application of more accurate workflows would require longer computing times with respect to docking. This is especially true considering the recent contributions provided by high performance computing systems to molecular docking, which enable the screening of millions of compounds in a reasonable time (Perez-Sanchez and Wenzel, 2011; Guerrero et al., 2012; Dong et al., 2018). However, future advances in hardware and software will help circumventing such limitation (De Vivo et al., 2016; Wang et al., 2018). Moreover, further advances in our ability to correctly estimate entropies of binding, which are usually not considered in the calculations, will certainly improve post-docking tools, and binding free energy predictions in general. The implementation of enhanced sampling MD protocols in post-docking protocols is another possibility that may enable a more efficient sampling of ligand-protein complexes. Because free energy predictions are heavily dependent on correct binding modes, this may have dramatic consequences on our ability to predict active ligands in virtual screenings. Another interesting question is how to further increase hit rates while enabling post-docking tools to identify significantly vs. moderately active hits. This is an important aspect that would make the subsequent hit-to-lead optimization much easier. Exponential consensus ranking approaches such as the one developed by Palacio-Rodríguez et al. (2019) could be of help, for example to favorably exploit both MM-PBSA and MM-GBSA ranked lists, which generally differ.

## Data Availability

The raw data supporting the conclusions of this manuscript will be made available by the authors, without undue reservation, to any qualified researcher.

## Author Contributions

GR conceived and wrote the study. LP contributed in writing and editing the manuscript.

### Conflict of Interest Statement

The authors declare that the research was conducted in the absence of any commercial or financial relationships that could be construed as a potential conflict of interest.
